# The Differentiation and Stress Response Factor XBP-1 Drives Multiple Myeloma Pathogenesis

**DOI:** 10.1016/j.ccr.2007.02.015

**Published:** 2007-04-10

**Authors:** Daniel R. Carrasco, Kumar Sukhdeo, Marina Protopopova, Raktim Sinha, Miriam Enos, Daniel E. Carrasco, Mei Zheng, Mala Mani, Joel Henderson, Geraldine S. Pinkus, Nikhil Munshi, James Horner, Elena V. Ivanova, Alexei Protopopov, Kenneth C. Anderson, Giovanni Tonon, Ronald A. DePinho

**Affiliations:** 1 Department of Medical Oncology, Dana-Farber Cancer Institute and Harvard Medical School, Boston, MA 02115, USA; 2 Center for Applied Cancer Science, Belfer Foundation Institute for Innovative Cancer Science, Dana-Farber Cancer Institute and Harvard Medical School, Boston, MA 02115, USA; 3 Departments of Medicine and Genetics, Dana-Farber Cancer Institute and Harvard Medical School, Boston, MA 02115, USA; 4 Department of Pathology, Brigham and Women's Hospital, Harvard Medical School, Boston, MA 02115, USA; 5 The Jerome Lipper Multiple Myeloma Center, Department of Medical Oncology, Dana-Farber Cancer Institute, Boston, MA 02115, USA

**Keywords:** CELLCYCLE

## Abstract

Multiple myeloma (MM) evolves from a highly prevalent premalignant condition termed MGUS. The factors underlying the malignant transformation of MGUS are unknown. We report a MGUS/MM phenotype in transgenic mice with *Eμ*-directed expression of the XBP-1 spliced isoform (XBP-1s), a factor governing unfolded protein/ER stress response and plasma-cell development. Eμ-XBP-1s elicited elevated serum Ig and skin alterations. With age, *Eμ-xbp-1s* transgenics develop features diagnostic of human MM, including bone lytic lesions and subendothelial Ig deposition. Furthermore, transcriptional profiles of *Eμ-xbp-1s* lymphoid and MM cells show aberrant expression of known human MM dysregulated genes. The similarities of this model with the human disease, coupled with documented frequent XBP-1s overexpression in human MM, serve to implicate XBP-1s dysregulation in MM pathogenesis.

## SIGNIFICANCE

**The significance of this work is underscored by the presence of MGUS in 1%–10% of adults, with 1%–3% progressing to MM annually. The genesis and progression of the disease are poorly understood, and the generation of a mouse model recapitulating many aspects of MGUS and MM provides an opportunity for the systematic dissection of the biological processes underlying disease progression. In particular, this genetically engineered MM-prone model enables validation of emerging MM-relevant (epi)genetic alterations and guides development of effective therapies targeting such validated rate-limiting aberrations. This genetic study and analysis of clinical samples suggest a pathogenetic role for sustained XBP-1s expression in the neoplastic transformation of plasma cells, indicating that chronic cellular stress contributes to the development of this common neoplasm.**

## Introduction

Multiple myeloma (MM) is a multifocal plasma-cell neoplasm characterized by serum monoclonal gammopathy and focal skeletal lesions. MM is preceded by a premalignant condition, *m*onoclonal *g*ammopathy of *u*ndetermined *s*ignificance (MGUS), present in 1% of adults over the age of 25 and rising to 10% prevalence in individuals in their tenth decade. The genetic and environmental factors underlying MGUS and its progression to MM are largely unknown, although aberrant physiological responses to chronic immune stimulation are among the number of potential pathogenetic associations (Sirohi and Powles, 2006). MM remains incurable despite conventional high-dose chemotherapy and transplantation, translating into a median survival of 6 years (Bergsagel and Kuehl, 2005; Mitsiades et al., 2004).

Extensive genomic analysis has revealed a large number of genes and loci associated with the development of MGUS and MM. Prominent cytogenetic events include chromosomal translocations involving Ig loci juxtaposed to the genes *Cyclin D1*, *FGFR3*, *MMSET*, and *c-MAF*, driving their overexpression. Additional genetic events typically associated with disease progression include *Ras* and *FGFR3* activating mutations; *c-MYC* deregulation; *p16INK4A*, *p18INK4C*, *TP53*, and *PTEN* tumor suppressor gene inactivation; and chromosome 13 deletion (Bergsagel and Kuehl, 2005; Kuehl and Bergsagel, 2002). Recent genomic studies have provided evidence of many additional genetic lesions driving human MM pathogenesis (Carrasco et al., 2006). The availability of a MM mouse model would facilitate the identification and validation of these MM-relevant genes and provide a preclinical model for assessing therapeutic agents directed against such targets.

Many experimental efforts to generate mouse models of B cell neoplasms, including MM, have typically involved targeted oncogene expression in the B cell compartment by transgenic and knockin approaches, alone or together with various tumor suppressor gene mutations (Cheung et al., 2004; Park et al., 2005). These modeling strategies have generally yielded B cell malignancies displaying immature phenotypes or plasmacytomas rather than classical MM. It is worth noting that mice do possess the inherent capacity to develop a spontaneous condition similar to human MGUS and MM, as evidenced by the capacity of the C57BL/KaLwRij strain to develop a plasma-cell dyscrasia, monoclonal gammopathy, and bone lytic lesions, albeit with late onset (after 2 years), low incidence (0.5%), and a propensity of these malignant plasma cells to home to lymphoid tissues other than the bone marrow (Garrett et al., 1997). Furthermore, the intravenous transplantation of these myeloma cells into syngeneic hosts has generated a single cell-line model that generates characteristic myeloma bone disease (Garrett et al., 1997). Along the lines of disease representation, it is worth noting that human MM consists of a minimum of four molecular subtypes (Carrasco et al., 2006) and that available human MM cell lines only partially represent these disease categories (D.R.C., G.T., and R.A.D., unpublished data). Together, these observations underscore the need for the continued development of genetic and cell-line models that capture the full range of genetic and biological diversity of human MM.

Based upon the above efforts to construct MM mouse models, we hypothesized that enforced B cell lineage-directed transgene expression of factors driving plasma-cell differentiation, alone or together with classical myeloma genes, would enhance the development of a MM-like disease. XBP-1 is a basic-region leucine zipper (bZIP) transcription factor of the CREB-ATF family and a major regulator of the unfolded protein response (UPR) and plasma-cell differentiation. XBP-1-deficient embryos die in utero from severe liver hypoplasia and resultant fatal anemia. Viable chimeras derived from XBP-1 null ES cells injected into Rag2 blastocysts reveal that XBP-1-deficient B cells proliferate and form germinal centers, yet there is a profound impairment in Ig secretion and absence of plasma cells (Reimold et al., 2001). XBP-1 is subject to alternative RNA processing, generating two mRNA transcripts encoding the same N-terminal DNA binding domain, but different C-terminal transactivation domains. The shorter spliced transcript, designated XBP-1s, possesses enhanced transactivation potential and stability relative to the product of the unspliced transcript, designated XBP-1u (Iwakoshi et al., 2003b; Lee et al., 2002; Shen et al., 2001). Thus, XBP-1u has no appreciable transactivation potential and may function as a dominant negative of XBP-1s (Lee et al., 2003).

Recent studies have uncovered several functions for XBP-1 and have implicated XBP-1 overexpression in human carcinogenesis and tumor growth under hypoxic conditions. Specifically, elevated XBP-1 mRNA levels have been detected in hepatocellular carcinomas (Lee et al., 2002) and in primary ERα-positive breast tumors (Fujimoto et al., 2003; Iwakoshi et al., 2003a). With regard to MM, abundant expression of XBP-1 has been detected in human MM cells (Munshi et al., 2004) and can be induced by IL-6, a growth factor for malignant plasma cells (Wen et al., 1999). However, these studies did not provide definitive documentation of the particular XBP-1 isoform preferentially produced in human MM or provide insights into the pathophysiological relevance of these XBP-1 isoforms in MGUS and MM (Davies et al., 2003; Munshi et al., 2004).

In this study, we have explored the biological impact of sustained XBP-1s expression in the lymphoid system, anticipating that this genetic event would be a necessary component along with other MM-relevant oncogenes and tumor suppressor gene manipulations to generate a MM-prone mouse model. Unexpectedly, XBP-1s overexpression alone yielded a MGUS-MM disease bearing many features that are classic hallmarks of the human disease on the clinical, pathological, and molecular levels. These genetic observations were bolstered by an analysis of clinical samples documenting frequent XBP-1s overexpression in human MM samples relative to normal plasma cells, together implicating XBP-1s dysregulation in the genesis of this malignancy. This murine model of MGUS-MM provides a framework for understanding the molecular and biological mechanisms governing the genesis and progression of these common and enigmatic plasma-cell dyscrasias.

## Results

### XBP-1 Expression in Human Normal Plasma Cells and MM Cells

**Figure 1:**
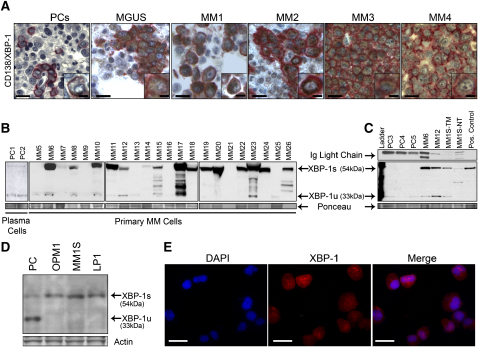
Predominance of the Spliced Form of XBP-1 over the Unspliced Form in MM Primary Tumors (A) Immunohistochemical analysis for XBP-1 expression was performed on bone marrow tissue microarrays from healthy (PCs) donors (n = 10) and MGUS (n = 20) and MM (n = 70) patients. Representative results are shown. CD138 is stained in red, and XBP-1 is stained in brown. Scale bars, 50 μm (10 μm in insets). (B) Plasma cells were isolated from the bone marrow of healthy donors (lanes 1 and 2) and MM primary tumors (lanes 3–24) using CD138 magnetic beads, and total protein extract (40 μg per lane) was subjected to western blot analysis using anti-XBP-1 antibodies. (C)Total protein extract (20 μg) from CD138 purified normal plasma cells (PC), MM primary tumors (MM), the MM1S MM cell line treated with 10 μg/ml of Tunicamycin (TM) and untreated (NT) for 4 hr, and 293T cells lentivirally infected with XBP-1s (Pos. Control) were electrophoresed and subjected to western blot analysis using XBP-1 antibodies. Protein loading was assessed by Ponceau staining and Ig light-chain immunostaining. (D) Total protein extracts (40 μg per lane) from CD138-purified normal plasma cells (PC) and MM cell lines were subjected to western blot analysis using XBP-1 antibodies. (E) Immunofluorescence analysis of XBP-1 expression on MM1S cell line. Scale bars, 50 μm.

The unanticipated MM-prone condition in our mouse model (see below) and previous studies documenting increased XBP-1 expression in human MM (Davies et al., 2003; Munshi et al., 2004) prompted detailed XBP-1 expression studies in clinical samples. Tissue microarray immunohistochemical (TMA-IHC) and western blot assays were used to examine the relative levels and patterns of XBP-1 and its isoforms in normal human plasma cells, MGUS, and MM. As shown in Figure 1A, TMA-IHC analysis of bone marrow tissues derived from healthy donors and from MGUS and MM patients revealed low or undetectable XBP-1 protein expression in the plasma cells from ten healthy donors yet robust and widespread XBP-1 expression in 10/20 (50%) of MGUS and 50/70 (70%) of MM samples. Consistent with the IHC data, western blot analysis of protein extracts from 22 samples of CD138^+^ MM cells demonstrated a prominent shift toward increased XBP-1s expression in the majority of MM primary tumor samples relative to normal plasma cells (Figures 1B and 1C), a pattern also evident in MM cell lines (Figures 1C–1E). These correlative translational observations and the known capacity of XBP-1 to drive expression of MM-promoting factors such as IL-6 (Iwakoshi et al., 2003b and see below) prompts speculation that chronically increased levels of XBP-1s may contribute to the development of human MGUS and MM.

### Generation of *Eμ-xbp-1s* Transgenic Mice Expressing XBP-1s in B Cells and Plasma Cells

**Figure 2:**
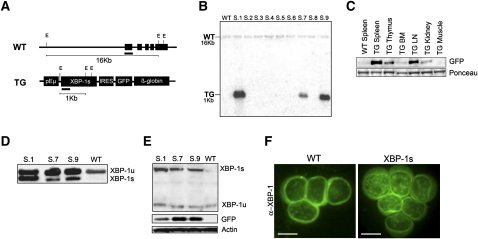
Generation and Characterization of *Eμ-xbp-1s* Transgenic Mice (A) Transgenic construct. The *xbp-1s* cDNA encoding the spliced form of mouse XBP-1 was cloned downstream of the immunoglobulin V_H_ promoter and Eμ enhancer (pEμ) elements. In addition to expressing XBP-1s, the transgene also encodes a farnesylated eGFP coding sequence behind an IRES element. (B) Southern blot genotyping of *Eμ-xbp-1s* transgenic mice was done on EcoRI-digested genomic tail DNA and hybridized with a radiolabeled probe encoding 5′ sequences from the mouse *xbp-1s* cDNA. Note genomic *xbp-1* sequences (WT) as well as transgenic sequences (TG). Only three of nine founders are shown. (C) High levels of transgene expression in spleen, lymph nodes, and thymus as evaluated by eGFP western blot analysis on total organ protein extracts isolated from 6-week-old control and transgenic mice. (D) RT-PCR analysis using mRNA from purified mouse B cells from *Eμ-xbp-1s* transgenic (S.1, S.7, S.9) and control (WT) mice. The 171 bp and 145 bp DNA fragments correspond to unspliced and spliced *xbp-1* mRNAs, respectively. (E) Western blot analysis of *Eμ-xbp-1s* transgenic (S.1, S.7, and S.9) and control (WT) splenic B220^+^ B cells. (F) Immunofluorescence staining of control (WT) and *Eμ-xbp-1s* transgenic splenic B cells. Scale bars, 10 μm. The genomic DNA, mRNA, and protein extracts were isolated from 6-week-old transgenic and control littermates.

On the basis of XBP-1s prominence in human MM and its potent transactivation potential (Iwakoshi et al., 2003a), we generated and characterized transgenic mice engineered to express the *xbp-1s* open reading frame under the control of the immunoglobulin *V_H_* promoter and *Eμ* enhancer elements (Eμ) (Figure 2A). Nine founders were obtained for the *Eμ-xbp-1s* transgene, and Southern blot comparison of the endogenous (WT) and transgenic *xbp-1s* hybridization signals (TG) indicated transgene copy numbers ranging from approximately 1 to 15. The Southern blot shown in Figure 2B documents three of the nine founders in one representative litter derived from C57BL/6-microinjected oocytes (Figure 2B; other founder blots not shown). Two independent founder lines, S.7 and S.9, and their transgenic progeny, were propagated and used in this study.

In accord with the known capacity of Eμ regulatory elements to drive transgene expression throughout B cell development, as well as in some T cells (Kemp et al., 1980), robust *Eμ-xbp-1s* transgene expression was detected in the spleen, lymph nodes, thymus, and bone marrow of all nine founders, including S.7 and S.9 progeny, as determined by eGFP expression (Figure 2C, only S.7 shown). Analysis of purified splenic B cells using the pan-B cell marker B220 from 6-week-old *Eμ-xbp-1s* transgenic and control mice showed increased levels of the spliced *xbp-1s* transcript in the transgenic mice correlating with transgene DNA copy number (Figure 2D and data not shown). Correspondingly, western blot analysis of B220^+^ splenic B cells confirmed elevated XBP-1s protein levels (Figure 2E), and anti-XBP-1 immunofluorescence showed stronger nuclear staining in *Eμ-xbp-1s* transgenic B cells compared with nontransgenic B cell controls (Figure 2F).

Together, these assays documented increased XBP-1s protein levels in the murine B lineage, enabling an in vivo analysis of the functional impact of enforced XBP-1s expression in B cell and plasma-cell compartments. To that end, all nine founders and the S.7 and S.9 progeny were assessed clinically using histological and flow cytometric analysis of lymphoid cells. While detailed phenotypic analysis described below focused on the two independent transgenic lines, S.7 and S.9, and their progeny, the same phenotype was observed in all nine founders.

### *Eμ-xbp-1s* Transgenic Mice Show Cutaneous and Renal Pathologies Resembling Patients with Chronic Plasma-Cell Disorders

**Figure 3:**
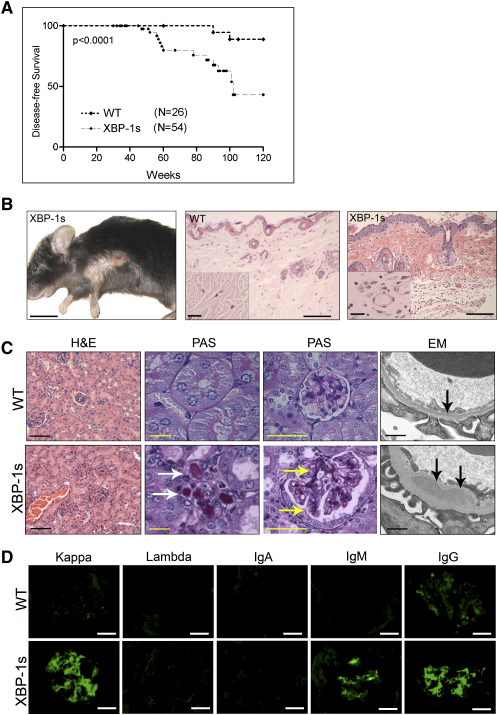
Survival, Skin, and Renal Alterations in *Eμ-xbp-1s* Transgenic Mice (A) Disease-free survival (lack of skin alterations) in *Eμ-xbp-1s* transgenic mice. Statistically significant differences (p < 0.0001) were detected between *Eμ-xbp-1s* transgenic and control littermates. (B) Skin alterations in *Eμ-xbp-1s* transgenic mice. Note loss of hair and skin thickening in axillary region. Histological H&E sections showing dermal changes in *Eμ-xbp-1s* transgenic mice. Note epidermal thickening, dermal fibrosis, and vascular proliferation (insets). Scale bars, 1.0 cm (left panel), 50 μm (middle and left panels), and 20 μm (insets). (C) Renal tissue sections from 40-week-old control (WT) and *Eμ-xbp-1s* transgenic mice were stained with H&E, PAS, or subjected to electron microscopic analysis (EM). White arrows, tubular protein deposition; yellow arrows, mesangial protein deposition; black arrows, subendothelial deposits. Scale bars, 50 μm (H&E), 20 μm (PAS), 0.1 μm (EM). (D) Glomerular immunoglobulin deposition. Serial frozen renal sections from control (WT) and *Eμ-xbp-1s* tissue sections were analyzed by immunofluorescence using specific antibodies for mouse immunoglobulin kappa and lambda light chains, as well as IgA, IgG, and IgM heavy chains. Scale bars, 20 μm.

Through 20 weeks of age, *Eμ-xbp-1s* transgenic mice exhibited normal gross appearance, behavior, and weight curves. Histological surveys of the major organs, as well as flow cytometric profiles of spleens using the lymphoid cells markers CD3 and B220, did not demonstrate any abnormalities (data not shown). By approximately 40 weeks of age, *Eμ-xbp-1s* transgenic animals began to manifest phenotypic changes in the skin and kidneys. *Eμ-xbp-1s* transgenic mice exhibited an overall shortened life span resulting from severe cutaneous disease and/or myeloma (Figure 3A; see below). Sixty percent of *Eμ-xbp-1s* transgenic mice developed hair loss and skin thickening around axillary and neck regions, whereas control littermates were unaffected (Figure 3B and data not shown). This cutaneous presentation was observed in all independently derived *Eμ-xbp-1s* transgenic lines. Histological examination of the *Eμ-xbp-1s* skin revealed epidermal thickening and some degree of hyperkeratosis with follicular plugging as well as dermal fibrosis with mild lymphoplasmacytic infiltrates and vascular hyperplasia. It is notable that these pathological changes are similar to those encountered in patients with specific autoimmune conditions and a rare plasma-cell disorder, termed POEMS (for *p*olyneuropathy, *o*rganomegaly, *e*ndocrinopathy, serum *m*onoclonal protein, and *s*kin lesions) (Dispenzieri, 2005) (Figure 3B).

By 40 weeks, *Eμ-xbp-1s* transgenic mice also showed renal pathology, including tubular cast deposition (Figure 3C, white arrows) and glomerular changes characterized by mesangial widening and deposition of PAS-positive material (Figure 3C, yellow arrows). Transmission electron microscopy showed mesangial and subendothelial deposition of electron-dense material (Figure 3C, black arrows). These renal lesions are similar to the pathologic manifestations present in human MM and other plasma-cell disorders with systemic chronic Ig overproduction and accumulation of light chains (light-chain cast nephropathy), paraproteins, and other Ig fragments (Herrera et al., 2004). To assess whether these lesions were Ig deposits, immunofluorescence with antibodies specific for light- and heavy-chain Ig was performed. Nonspecific trapping of light- and heavy-chain Ig was present and consisted of either polyclonal heavy and light chains or clonal IgM or IgG heavy chain and kappa light chains (Figure 3D and data not shown). To determine whether amyloid depositions were present, a feature occasionally seen in MM patients, Congo red stains performed across several tissues and independent cell lines were negative (see Figure S1A in the Supplemental Data available with this article online) suggesting that amyloid deposition, which is observed rarely (5%–15%) in human MM patients (Kyle and Gertz, 1990), is not a feature of the *Eμ-xbp-1s* disease model.

### Altered Proliferative Potential of Splenic B Cells and Hypergammaglobulinemia in *Eμ-xbp-1s* Transgenic Mice

Serial gross, histological, and flow cytometric examination of the spleen and bone marrow of *Eμ-xbp-1s* transgenic mice showed alterations after 20–40 weeks of age. The spleens appeared slightly enlarged and showed increased plasma cells around periarterial sheaths (Figure S1B, arrows). Flow cytometric analysis showed unaltered total numbers of T cell (CD3) and B cell (B220) populations in *Eμ-xbp-1s* transgenic spleens (Figure S2A). The total number of mononuclear cells between wild-type (8.05 × 10^7^ ± 0.5) and *Eμ-xbp-1s* transgenic (7.95 × 10^7^ ± 1.5) spleens did not differ. *Eμ-xbp-1s* transgenic mice at 40 weeks of age showed a slightly enlarged population of CD23^+^ cells (49.1% ± 3.9% versus 40.2% ± 0.1%) and a reciprocal decrease in CD23^−^ cells (43.3% ± 1.5% versus 53.4% ± 1.9%). We observed a trend toward increased number of precursor and mature follicular cells T2/M cells (CD23^+^/CD21^+^/IgM^+^ cells) (34.1% ± 1.2% versus 26.4% ± 1.4%) as well as marginal zone (MZ) B cells (CD23^−^IgM^hi^CD21^+^ cells) (3% ± 0.1% versus 1% ± 0.0%) in the spleens of *Eμ-xbp-1s* transgenic mice (Figure S2B). In addition, the fraction of B220^+^ cells in the bone marrow was significantly increased in *Eμ-xbp-1s* transgenic mice with an increase in the mature IgM^+^B220^+^ B cell population (17.2% ± 1.2% versus 10.8% ± 0.2%) and a relative decrease of pro-B cells (B220^+^CD43^+^) in relation to pre-B cells (B220^low^CD43^−^) in *Eμ-xbp-1s* transgenic bone marrows (Figure S2B).

Overall, these studies reinforce the view that enforced XBP-1s expression has an impact beyond the well-established role of this transcription factor in the terminal differentiation of B cells into plasma cells (Shaffer et al., 2004).

**Figure 4:**
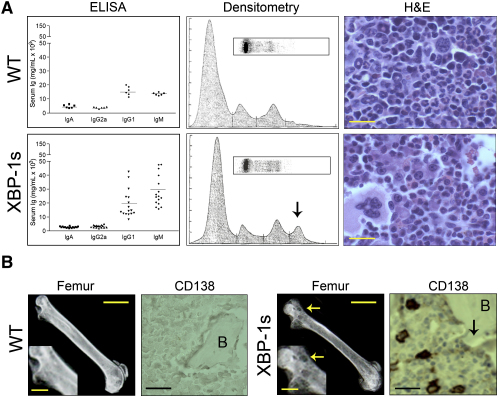
Hypergammaglobulinemia, Bone Marrow Plasmacytic Infiltrates, and Bone Lytic Lesions in *Eμ-xbp-1s* Transgenic Mice (A) Marked elevation of serum immunoglobulin levels in *Eμ-xbp-1s* transgenic mice. Serum plasma from control (WT) and *Eμ-xbp-1s* transgenic mice were measured by ELISA, protein serum electrophoresis, and densitometry. Note the presence of M spike (arrow) in *Eμ-xbp-1s* transgenic mice. Representative bone marrow biopsies from control and *Eμ-xbp-1s* transgenic mice were analyzed by light microscopy (H&E) to reveal increased plasma-cell infiltrates in the marrow of *Eμ-xbp-1s* transgenic mice. (B) Bone lytic lesions in *Eμ-xbp-1s* transgenic mice. Femurs from control (WT) and *Eμ-xbp-1s* transgenic mice were dissected to detect the presence of bone lytic lesions by X-ray (white arrows) and immunohistochemical analysis (black arrow). B, cortical bone. Scale bars 20 μm (upper panel), 0.5 cm (lower panels, femur), 0.25 cm (insets), 50 μm (CD138).

**Figure 5:**
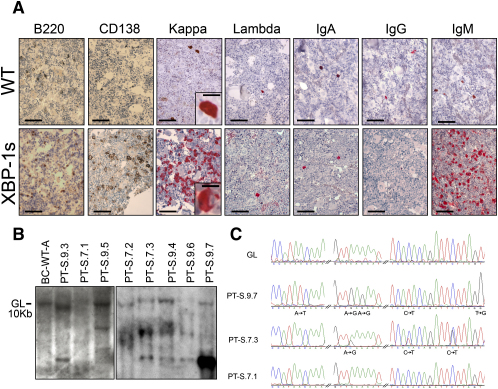
Evidence of Clonality and Hypermutation of Expressed Ig Genes in *Eμ-xbp-1s* Transgenic Mice (A) IHC analysis of plasma-cell clonality from bone marrow biopsies. Scale bars, 50 μm (10 μm in insets). (B) Southern blot analysis for clonotypic immunoglobulin heavy-chain rearrangement in *Eμ-xbp-1s* myeloma tumors. High-molecular-weight DNA was isolated from snap-frozen control or myeloma marrows, digested with EcoRI restriction enzyme, and hybridized with a  1.9 BamHI-EcoRI genomic probe fragment downstream and adjacent to the mouse heavy-chain locus JH segment. GL denotes the germline band. (C) DNA sequence chromatograms of PCR products detect mutations in the 3′ JH4 region of rearranged variable (V) genes in *Eμ-xbp-1s* tumors.

Consistent with the above observation of expanded B cell compartment, plasma immunoglobulin levels of both IgM and IgG types by ELISA were significantly increased in *Eμ-xbp-1s* transgenic mice (Figure 4A). Serum protein electrophoresis (SPEP) revealed presence of an M spike (Figure 4A, arrow) in the majority of *Eμ-xbp-1s* transgenic mice, but not in control littermates as early as 20 weeks of age—a feature that increased in frequency and magnitude with advancing age (data not shown). Notably, these serum changes were associated in some cases with bone lytic lesions and increased numbers of plasma cells in the bone marrow that varied from 5%–30% of the total bone marrow cellularity compared with <5% for nontransgenic controls (Figures 4B and 5A; Table S1).

**Figure 6:**
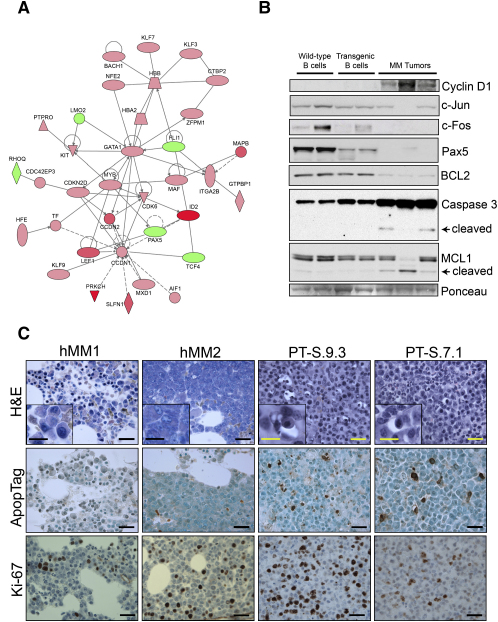
Altered Gene Expression of *Eμ-xbp-1s* B Cells and Tumor Plasma Cells and Increased Apoptosis of Tumor Plasma Cells (A) Ingenuity analysis showing altered expression of Cyclin D1 and c-MAF in myeloma tumors. In red are significantly overexpressed genes and in green are downregulated genes. Color intensity is proportional to the SAM score (Tusher et al., 2001). (B) Western blot analysis showing activation of caspase-3, as well as decreased c-JUN, c-FOS, MCL1, and BCL2 in transgenic B cells and MM tumors as compared to wild-type samples. (C) IHC showing increased apoptosis (Apoptag) and proliferation index (Ki-67) in *Eμ-xbp-1s* myeloma primary mouse tumors (PT) as compared with human myeloma tumors (hMM). Scale bars, 50 μm (20 μm in insets).

On the molecular level, transcriptome comparison of *Eμ-xbp-1s* and nontransgenic B cells (n = 5 mice each) by *s*ignificance *a*nalysis of *m*icroarrays (SAM) (Tusher et al., 2001) revealed 1119 genes that were differentially expressed (false discovery rate [FDR] = 1%), 808 upregulated and 311 downregulated in *Eμ-xbp-1s* B cells versus nontransgenic B cells. Among the genes selectively altered in *Eμ-xbp-1s* B cells are those with known relevance to human MM pathogenesis, such as Cyclin D1, Cyclin D2, MAF, and MAFB (Figure 6A and data not shown) (Kuehl and Bergsagel, 2002; Shaffer et al., 2004). Given the role of these genes in cell-cycle regulation, proliferative potential of wild-type and transgenic B cells were examined ex vivo. Consistent with the molecular changes, *Eμ-xbp-1s* transgenic B cells exhibited increased incorporation of thymidine relative to nontransgenic controls (Figure S2C, p = 0.002). Enhanced proliferative potential of the transgenic B cells was also observed in response to stimulation with B cell activators CD40, anti-IgM, or CD40 together with anti-IgM (data not shown).

Knowledge-based pathway analysis (Calvano et al., 2005) of the 1119 SAM-significant genes also revealed alterations in several pathways (p < 0.0001). A notable MM-relevant pathway that was prominently altered is IL-6, where the two components of the IL-6 receptor, IL-6R and gp130, show significant upregulation, along with three members of the CEBP family, CEBPA, CEBPB, and CEBPD, in addition to several downstream targets of these transcriptional factors, such as ALOX5, LITAF, MMP8, and IFITM3 (Figure S3A). Prompted by this observation, we examined the level of serum IL-6, to determine whether a more general dysregulation of this pathway was present in these mice. Consistent with the activation of this pathway, serum IL-6 levels were increased in *Eμ-xbp-1s* transgenic mice relative to control littermates (Figure S2C, p < 0.005), while the plasma levels of other cytokines, including IL-2, IL-4, IL-5, and IL-10, were unchanged (data not shown).

Taken together, the phenotypic and molecular analyses of transgenic and nontransgenic B cells uncovered cancer-relevant biological consequences of XBP-1s overexpression, including B cell hyperproliferation and IL-6 activation, both of which are plausible mechanisms capable of driving plasma-cell transformation.

### Spontaneous MGUS and MM in Aging *Eμ-xbp-1s* Transgenic Mice

The above constellation of findings prompted detailed analysis of aging *Eμ-xbp-1s* and nontransgenic controls for evidence of MGUS and/or MM. As early as 40 weeks of age, a classical MGUS profile emerged only in the *Eμ-xbp-1s* transgenic mice. Between 11 and 20 months of age, approximately 26% (9/35) of *Eμ-xbp-1s* transgenic mice showed a clonal M spike in the serum and expanded populations of clonal plasma cells in the bone marrow (<10% of the total bone marrow mononuclear cells), without bone lytic lesions and consistent with a pattern of MGUS (Table S1; data not shown). Between 14 and 24 months of age, 26% (8/35) of *Eμ-xbp-1s* transgenic mice progressed to frank MM as defined by a clonal M spike in the serum, bone marrow consisting of >10% of clonal plasma cells, and associated bone lytic lesions on radiographic examination (Figures 4A, 4B, and 5A; Table S1; and data not shown). Clonality of the MM cells was evidenced by IHC of immunoglobulin heavy and light-chain subtype expression, as performed in the clinical laboratory (Figure 5A), and by Southern blot analysis of *IgH* gene rearrangement analysis in bone marrow plasma cells (Figure 5B). In accord with the concept of a postgerminal center cell derivation of human MM cells (Kuehl and Bergsagel, 2002), some of the *Eμ-xbp-1s* MMs showed evidence of somatic hypermutation between the body of the 3′ JH4 region of rearranged variable (V) genes (Figure 5C). Neither MGUS nor MM was detected in control littermates at any age, indicating that XBP-1s overexpression promotes the development of a condition similar to MGUS and MM. In summary, spontaneous progression from MGUS to MM in the *Eu-xbp-1s* transgenic mice clinically mirrors disease progression in the human.

To further compare this mouse model with the human disease, we performed transcriptome profiling to define the *Eμ-xbp-1s* myeloma signature. Transcriptome analyses in the mouse identified 708 genes overexpressed and 1784 genes downregulated (FDR = 1%) in *Eμ-xbp-1s* MM cells relative to B cells from young neoplasm-free *Eμ-xbp-1s* mice. Supporting a pathogenetic role for XBP-1s overexpression in the myeloma genesis in this model, several known MM genes that were dysregulated in premalignant *Eμ-xbp-1s* B cells exhibited similar patterns of alterations in XBP-1s MM cells, including Cyclin D1, MAF, CEBPA, CEBPB, CEBPD, IL6ST (upregulated), and FOS (downregulated). Conversely, some notable MM signature genes were found to be selectively dysregulated in *Eμ-xbp-1s* MM, but not in *Eμ-xbp-1s* B cells, in line with the need for additional spontaneously occurring cooperating events as reflected in the long latency of the MM phenotype. Among such genes selectively upregulated in the tumors were APRIL and BAFF, both of which have been found to be overexpressed in various B cell malignancies, including MM (He et al., 2004; Novak et al., 2004). However, unlike the human counterpart, the *Eμ-xbp-1s* MM transcriptome revealed evidence of prominent activation of proapoptotic tumor suppression mechanisms characterized by downregulation of the antiapoptotic genes, MCL1 and BCL2, which are typically upregulated or amplified in aggressive human MM cases (Carrasco et al., 2006; Le Gouill et al., 2004; Mitsiades et al., 2004). Interestingly, MCL1 and BCL2 were not downregulated in hyperproliferative premalignant transgenic B cells (Figure S3B and data not shown). Consistent with the *Eμ-xbp-1s* gene expression pattern and in contrast to the human disease, we documented a marked increase in apoptosis and proliferation in murine MM samples relative to those in human MM samples (Figures 6B and 6C). Such observations gain added significance in light of the observation that MCL1 maps to a region of gene amplification in aggressive human MM primary tumors and prompt speculation that MCL1 and BCL2 play critical roles in the progression of human myeloma (Carrasco et al., 2006; Le Gouill et al., 2004; Mitsiades et al., 2004)—a theory that can now be tested in this genetic model system.

## Discussion

In this study, we provide genetic evidence and correlative human data supporting a role for the differentiation and unfolded protein/ER stress response factor X box binding protein-1 spliced isoform (XBP-1s) in the development of MGUS and MM. Our molecular and phenotypic analyses of the XBP-1s-driven myeloma model implicate the chronic ER stress response in MM pathogenesis.

### A Genetically Engineered Mouse Model of Human MM

In this *Eμ-xbp-1s* transgenic model, enforced XBP-1s expression in the B cell compartment enhances B cell proliferative potential and activates known MM-relevant pathways, leading to the development of MGUS and MM disease possessing many of the molecular, cellular, and clinical features of the human condition. As in other genetically engineered mouse (GEM) models of human diseases, some differences do exist between the mouse and human conditions, particularly in the levels of genome instability. In addition, human MM is predominantly of IgG isotype, but *Eμ-xbp-1s* MGUS and MM cases are either the IgG isotype (50%) or the IgM isotype (50%). Another difference is the prevalence of dermatitis and splenic plasmacytosis in the mouse that, in humans, are more commonly encountered in patients with atypical plasma-cell malignancies such as the POEMS syndrome and Castleman's disease (Dispenzieri, 2005; Shahidi et al., 1995).

At the same time, there are remarkable similarities between the disease phenotypes in mice and humans. These include progression from MGUS to MM accompanied by bone marrow involvement with clonal plasma cells, serum M spike, bone lytic lesions, and Ig deposition in the kidney, all classical defining features of human MM. Molecularly, *Eμ-xbp-1s* MM exhibited dysregulation of several signature MM genes and pathways. For example, expression alteration of genes such as Cyclin D1, gp130, and MAF parallel the dysregulation of these genes identified in human MM cells, which are variously driven by recurrent chromosomal translocations, gene amplification, and/or transcriptional mechanisms and represent essential elements in the genesis and progression of human MM (Kuehl and Bergsagel, 2002). The proliferation effect induced by IL-6 is mediated by the PI3K and MAPK pathways and through the activation of CEBPB. Given the known role of the CEBP family of transcription factors in carcinogenesis (Sebastian and Johnson, 2006), the strong overexpression of CEBPB, as well as CEBPA and CEBPD, may contribute to the strong proliferative drive of *Eμ-XBP-1s* B transgenic cells that, through the acquisition of additional genetic/epigenetic lesions, could promote the development of MGUS and MM. Additionally, CEBPB exerts an inhibitory activity toward the AP1 complex, at both the expression and transcriptional levels (Gagliardi et al., 2003), demonstrated also in a human MM cell line (Takeshita et al., 2004), that may be responsible for the low JUN and FOS levels in the *Eμ-xbp-1s* B cells. Also, in accord with human MM, *Eμ-xbp-1s* MM cells exhibit overexpression of APRIL (*a pr*oliferation-*i*nducing *l*igand). APRIL is a member of the tumor necrosis family of ligands, which also includes B lymphocyte stimulator/B cell activating factor (BlyS/BAFF, which is also upregulated in *Eμ-xbp-1s* MM cells), and these factors have been shown to serve essential prosurvival roles in normal and malignant B lymphocytes. Increased expression of APRIL or BlyS have been reported in various B cell malignancies, including MM (He et al., 2004; Novak et al., 2004).

Taken together, these cross-species multilevel comparisons, spanning from molecular to organismal levels, provide a measure of validation that the *Eμ-XBP-1s* mouse represents a relevant model of human MM, suggesting its utility in the study of pathways known or presumed to be altered in the human disease. In this regard, molecular characterization of mouse and human MM has implicated two antiapoptotic genes, MCL1 and BCL2, in MM progression. MCL1 and BCL2 are commonly amplified and overexpressed in human MM (Carrasco et al., 2006; Le Gouill et al., 2004; Spets et al., 2002); however, they were downregulated in the *Eμ-xbp-1s* tumors (Figure 6B), but not in premalignant B cells. This pattern of expression is consistent with the observed increase in apoptosis in the *Eμ-xbp-1s* mouse MM when compared to human MM samples (Figure 6D), suggesting that active proapoptotic mechanisms may be acting to constrain the MM phenotype in this model. Moreover, analysis of published data comparing the expression level of plasma cells from healthy donors, MGUS, and MM patients (Zhan et al., 2002) indeed shows that only a subset of MM samples have significantly higher MCL1 levels than healthy donors, whereas MGUS patients and normal donors present comparable MCL1 expression (Figure S3B). These observations prompt speculation that MCL1 plays an especially critical role in the progression of human MM and motivate additional genetic modeling studies in *Eμ-xbp-1s* system to formally test the role of MCL1 in MM progression.

Given the relatively long latency and low penetrance in the appearance of MGUS and MM in this model, it stands to reason that additional genetic lesions and pathway dysregulations are needed to develop MGUS and MM in the *Eμ-xbp-1s* system. Indeed, several hallmark genes and pathways linked to MM pathogenesis are altered at the premalignant B cell level, for example Cyclin D1, Cyclin D2, MAF, and MAFB, providing a proliferation impetus to precursor B cells and plasma cells. However, other genes are consistently dysregulated specifically in the transition to MM, but not in *Eμ-xbp-1s* B cells or in the nontransgenic B cells such as BAFF, APRIL, and several genes associated in MM human studies with a high degree of proliferation and poor prognosis (TOP2A, BIRC5, CCNB2, NEK2, AURKA, BUB1, CDC2, and CDCA1; data not shown), suggesting that other genetic and/or epigenetic additional lesions are needed to drive the progression into MGUS and furthermore into MM. Therefore, *Eμ-xbp-1s* transgenic mice constitute a valuable tool for in vivo testing of genetic lesions capable of inducing MGUS and full-blown MM, on the backdrop of a paraphysiological increase in plasma-cell number.

### Role of XBP-1s and ER Stress Response in MM Pathogenesis

In humans, the *XBP-1* gene has not been implicated in MM pathogenesis because it does not reside in a region targeted from chromosomal amplification and/or translocation in human MM tumors (Bergsagel and Kuehl, 2005; Carrasco et al., 2006). However, there is evidence that genes affecting *XBP-1* processing are subject to genetic changes, potentially leading to epigenetic dysregulation of the XBP-1 network in MM. For example, the activating transcription factor 6α (ATF6α), S1P, and S2P proteases, as well as ER transmembrane protein kinase and endoribonuclease inositol-requiring enzyme-1α (IREα), are all members of the proximal transducers of the mammalian UPR (Ma and Hendershot, 2001). Furthermore, it is known that XBP-1 activation/overexpression is a downstream target (Gass et al., 2004) of the ER stress response pathway, which is perturbed in the setting of viral infections and inflammatory responses (Li et al., 2005). This gains significance in light of the long suspected link between autoimmunity and/or inflammation with MM (Sirohi and Powles, 2006) as well as our genetic observations that sustained XBP-1s overexpression alone is capable of driving MM development, raising the intriguing possibility that this physiological stress response could have unwanted “side effects” that are of fundamental relevance to human MM pathogenesis. In particular, we speculate that the long-term survival of plasma cells and MGUS cells may require enhanced activation of XBP-1s to handle the high levels of antibody production and/or environmental stresses. In this scenario, enhanced activation of XBP-1s may lead to chronic IL-6 production and activation of other MM-relevant circuits, ultimately driving progression along the MGUS-MM continuum.

In summary, we propose *Eμ-xbp-1s* transgenic mouse as a relevant model of human MGUS and MM. Additionally, this genetic study, coupled with analysis of human samples, highlights the potential importance of XBP-1s in the pathogenesis of human MGUS and MM.

## Experimental Procedures

### Transgenic Mice

The mouse *xbp-1s* cDNA (Calfon et al., 2002) was cloned into the pBluescript II vector, which also contains a 1 kb fragment of the mouse variable chain enhancer and promoter (pEμ) and a 1.6 Kb fragment of the human β-globin gene to provide introns and the polyadenylation signal sequence. The transgene also encoded farnesylated eGFP coding sequences behind an IRES element. The transgenic encoding sequences were released from the vector by BssHII digestion and purified by zonal sucrose gradient centrifugation and microinjected into C57BL/6 oocytes. Transgene-positive mice were mated with C57BL/6 mice to generate transgenic and control littermates on the same inbred C57BL/6 background for all subsequent analyses. Screening of the transgenic founders and copy-number analysis were done using Southern blot analysis with the entire *xbp-1s* cDNA as a radiolabeled probe. Subsequent screening of transgenic mice was done by PCR analysis using a set of GFP-specific primers (5′-ACGTAAACGG CCACAAGTTC-3′ and 5′-AAGTCGTGCT TCATGTG-3′). All animal experiments were approved by and conform to the standards of the Institutional Animal Care and Use Committee at the DFCI.

### Mouse B220^+^ and Human Plasma-Cell CD138^+^ Isolation, RNA Preparation, Expression Profiling, and Reverse Transcription PCR

B220^+^ splenic B cells were isolated using magnetic microbeads from Miltenyi Biotec. Plasma cells were isolated from human bone marrow mononuclear cells from healthy donors (Cambrex BioScience) using immunomagnetic bead selection with mouse anti-human CD138 monoclonal antibodies as described (Zhan et al., 2002), except that we used LS MACS separation columns (Miltenyi Biotec) and before incubating the bone marrow mononuclear cells to CD138-coated magnetic beads, we removed contaminating histiocytes and macrophages by preloading and washing out the cells in the magnetic columns and field. Total RNA was isolated from control and transgenic B220^+^ splenic mononuclear cells or myeloma tumors using TRIzol Reagent. Affymetrix 430 2.0 arrays were hybridized at the DFCI Microarray Core Facility. CEL files were obtained using Affymetrix Microarray Suite 5.0 software. The DNA Chip Analyzer (dChip) was used to normalize all CEL files to a baseline array with overall median intensity, and the model-based expression (perfect match minus mismatch) was used to compute the expression values. The same data were also analyzed using the Ingenuity Pathways Analysis (Ingenuity Systems) software. For SAM analysis, probes were included in the analysis if they fulfilled the following criteria (Tonon et al., 2005): (1) SD > 0.3; (2) >20% of samples with expression value > 100; (3) >20% of samples with presence call.

The primers for *xbp-1s* cDNA were 5′-ACACGCTTGG GAATGGACAC-3′ and 5′-CCATGGGAAG ATGTTCTGGG-3′, which flanked the spliced region in *xbp-1s*. PCR products were analyzed on a 3% agarose gel. The Institutional Review Board of Brigham and Women's Hospital approved the human research studies, and all subjects provided written informed consent approving use of their samples for research purposes.

### X-Ray, Histopathology, and Immunohistochemistry

Bone X-ray was performed using Faxitron X-ray Specimen Radiographic System and Kodak X-OMAT-TL films. Tissues were fixed, processed, sectioned, and stained with hematoxylin-eosin (H&E) or PAS for light microscopy examination by routine methods. Spine and femurs were additionally treated for 1 hr in decalcifying solution (Fisher Scientific). Immunohistochemistry was performed according to standard procedures. The anti-mouse XBP-1 rabbit polyclonal antibody (M-186) was obtained from Santa Cruz Biotechnology. The rat anti-mouse CD45R/B220 (RA3-6B2), rat anti-mouse CD138 (281-2), and mouse anti-human/mouse Ki-67 (B56) monoclonal antibodies were obtained from BD Biosciences. The primary antibodies were visualized with the corresponding biotinylated antibody coupled to streptavidin-peroxidase complex (Vector Labs). The goat anti-mouse polyclonal antibodies for immunoglobulin heavy chains IgA, IgM, IgG, and kappa or lambda light chains were all conjugated to horseradish peroxidase and obtained from Southern Biotechnology. All antibodies, conditions, and reactivities were tested in positive control slides. Apoptag assay (Chemicon) was performed on tissue slides according to the manufacturer's protocols.

### Cytospin Preparation, Immunofluorescence, and Transmission Electron Microscopy

Cytospin slides were prepared using a cytocentrifuge (Thermo Shandon), fixed 2 min at RT in acetone:methanol (1:1), and stained using anti-mouse XBP-1 rabbit polyclonal antibody as a primary antibody followed by visualization with fluorescein anti-rabbit IgG or directly labeled using fluorescein anti-mouse IgG (both from Vector Labs). Immunofluorescence was performed on murine kidney samples using fluorescein (FITC)-conjugated goat antibodies against mouse immunoglobulins IgA, IgG, IgM, kappa light chain, and lambda light chain (Southern Biotechnology). For kidney immunofluorescence, tissue sections were fixed in glutaraldehyde and processed using fluorescein-conjugated goat anti-mouse antibodies for immunoglobulin heavy chains IgA, IgM, IgG, and light chains kappa or lambda from Southern Biotechnologies. Transmission electron microscopy of murine kidney samples was performed using standard procedures after fixation in Karnovsky's media.

### Flow Cytometry

Red blood cells were removed from single-cell suspensions from spleens, thymuses, and bone marrow by ammonium chloride treatment (Sigma). Cells were then washed in 3% BSA in PBS and stained for 30 min on ice with a combination of the following antibodies: B220-FITC, B220-PE, CD3-PE, CD138-PE, CD8-FITC, CD4-PE, CD21-FITC, CD43-FITC (BD Biosciences), IgD-FITC, IgM-APC, and CD23-PE (eBioscience). Cells were washed twice and analyzed on a FACScalibur machine (Becton Dickinson) using cell Quest software (Becton Dickinson). An average of 10^5^ cells was recorded in each case.

### In Vitro Proliferation

B220^+^cells (2 × 10^5^) were plated in triplicate in 96-well plates before stimulation with LPS (10 μg/ml, Sigma), anti-mouse CD40 (1 μg/ml, BD Pharmigen), anti-IgM (10 μg/ml, BD Pharmigen), or CD40 and anti-IgM. After 16 hr of stimulation, the cells were pulsed with [^3^H]thymidine for the final 16 hr of growth before thymidine incorporation was measured by a scintillation counter. Results are expressed as mean ± SD of triplicate cultures. Similar results were obtained in three independent experiments.

### Clonality and Hypermutation Analysis

Southern analysis for clonotypic *IgH* rearrangements and hypermutation analysis of the 3′ JH4 region of rearranged variable (V) genes were performed as described previously (Carrasco et al., 2006; McDonald et al., 2003).

### Western Blots

Whole-cell extracts from B220^+^ splenocytes single- or snap-frozen tissues were prepared, electroblotted onto PVDF membranes (Amersham), and probed with primary antibodies according to standard procedures. The anti-mouse XBP-1 rabbit polyclonal antibody (M-186), anti-β-actin-HRP (C-11), c-FOS (4-10G), and anti-GFP (B-2) antibodies were from Santa Cruz. Cyclin D1 (DCS6), c-JUN (60A8), and caspase-3 (8G10) antibodies were obtained from Cell Signaling; MCL1 (Rockland), PAX5 (Chemicon), and BCL2 (3F11) antibodies from BD Biosciences were also used. Following incubation with horseradish peroxidase-conjugated goat anti-rabbit or anti-mouse secondary antibody (Pierce), bound immunoglobulins were detected using ECL detection solutions (Pierce). For consecutive staining with different antisera, membranes were stripped with Restore western blot stripping buffer (Pierce). Anti-β-actin immunoreactivity is included as a loading control.

### Chemokine, Immunoglobulin, and Paraprotein Determination

Serum chemokine and immunoglobulin concentrations were measured by ELISA using the Beadlyte mouse multicytokine detection system from Upstate. Elevated levels of IL-6 were confirmed using the Quantikine immunoassay from R&D Systems. All samples were analyzed in triplicate. Paraproteins (M spikes, extra gradients) were detected using Paragon SPE electrophoresis kit (Beckman Coulter Inc).

### Microarray Data

The raw data for expression profiling and the CEL files are available at Gene Expression Omnibus (http://www.ncbi.nlm.nih.gov/geo/) with accession number GSE6980.

## Accession Numbers

The raw data for expression profiling and the CEL files are available at Gene Expression Omnibus (http://www.ncbi.nlm.nih.gov/geo/) with accession number GSE6980.

## Supplemental Data

Document S1. Three Supplemental Figures and One Supplemental Table
