# Supplementary material for: The Differentiation and Stress Response Factor XBP-1 Drives Multiple Myeloma Pathogenesis
Source: Cancer Cell. 2007 Apr 10;11(4):349–60. doi: 10.1016/j.ccr.2007.02.015 (PMC1885943; doi:10.1016/j.ccr.2007.02.015)
Supplement: Document S1. Three Supplemental Figures and One Supplemental Table [file mmc1.pdf]

Cancer Cell, Volume *11*

Supplemental Data

The Differentiation and Stress Response Factor

XBP-1 Drives Multiple Myeloma Pathogenesis

Daniel R. Carrasco, Kumar Sukhdeo, Marina Protopopova, Raktim Sinha, Miriam Enos, Daniel E. Carrasco, Mei Zheng, Mala Mani, Joel Henderson, Geraldine S. Pinkus, Nikhil Munshi, James Horner, Elena V. Ivanova, Alexei Protopopov, Kenneth C. Anderson, Giovanni Tonon, and Ronald A. DePinho

Figure S1.

(A) *Eμ-xbp-1s* transgenic mice do not develop amyloid tissue deposition. Kidney tissue sections from a human patient with amyloidosis (left) and 40 week *Eμ-xbp-1s* transgenic mice (right) were stained with Congo Red and analyzed under light (upper) or dark field (lower) microscopy. Representative sections are shown. Note absence of apple green staining in *Eμ-xbp-1s* transgenic kidney. Scale bars, 20

μm.

(B) Increased numbers of plasma cells in *Eμ-xbp-1s* transgenic spleens. Spleens from 40-week-old control (WT) and *Eμ-xbp-1s* transgenic mice were analyzed by light microscopy (H&E) or immunostained with anti-CD138 antibodies, and by flow cytometric analysis on single cell suspensions with anti-B220 and anti-CD138 antibodies. Arrows indicate periaterial sheaths. Scale bars: H&E (left), 50 μm; H&E (right), 20 μm; CD138, 50 μm.

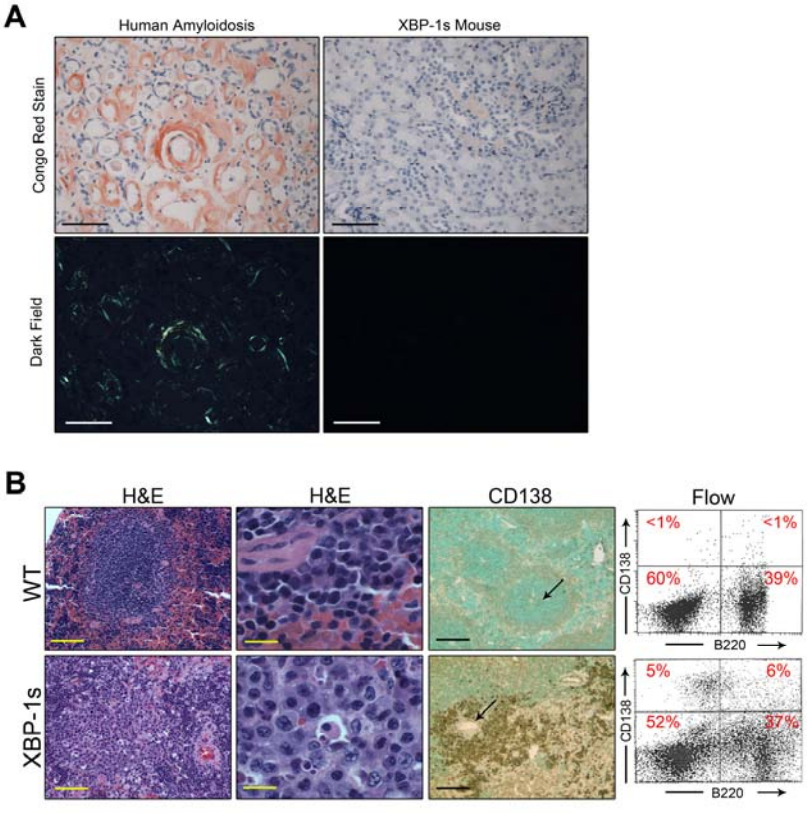

Figure S2.

(A) Unaltered total numbers of T-cell (CD3) and B cell (B220) populations in *Eμ-xbp-1s* transgenic spleens. Splenic B cells from 20-week-old control (WT) and *Eμ-xbp-1s* transgenic mice were double stained with anti-CD3 and B220 antibodies.

(B) Elevated splenic marginal zone B cells and mature follicular B cells and bone marrow mature B cells in *Eμ-xbp-1s* transgenic mice. Splenic B cells from 20-week-old nontransgenic (WT) and *Eμ-xbp-1s* transgenic mice were double stained with anti-CD3 and B220 antibodies or triple stained with anti-IgM, CD21, and CD23 before FACS analysis to distinguish MZ and T2/M populations after gating on $CD23^-$ and $CD23^+$ cells. Bone marrow cells from 20-week-old mice were labeled with anti-B220 and CD43 antibodies to characterize pro- and pre-B cells, as well as with anti-B220 and IgM to characterize mature B cells present in the bone marrow. The flow analysis were done for two independent animals and repeated twice. Representative selected profiles are shown.

(C) Increased serum levels of IL-6 and proliferation of $B220^+$ B cells in *Eμ-xbp-1s* transgenic mice. The plasma levels of IL-6 were analyzed in control (WT) and *Eμ-xbp-1s* transgenic mice using ELISA assay (left panel). Enriched spleen B cell populations obtained from control and *Eμ-xbp-1s* transgenic mice were cultured in the absence or presence of LPS, and proliferation was assayed by measuring incorporation of [^{3}H]thymidine. Each bar represents the mean of triplicates. Error bars show SD.

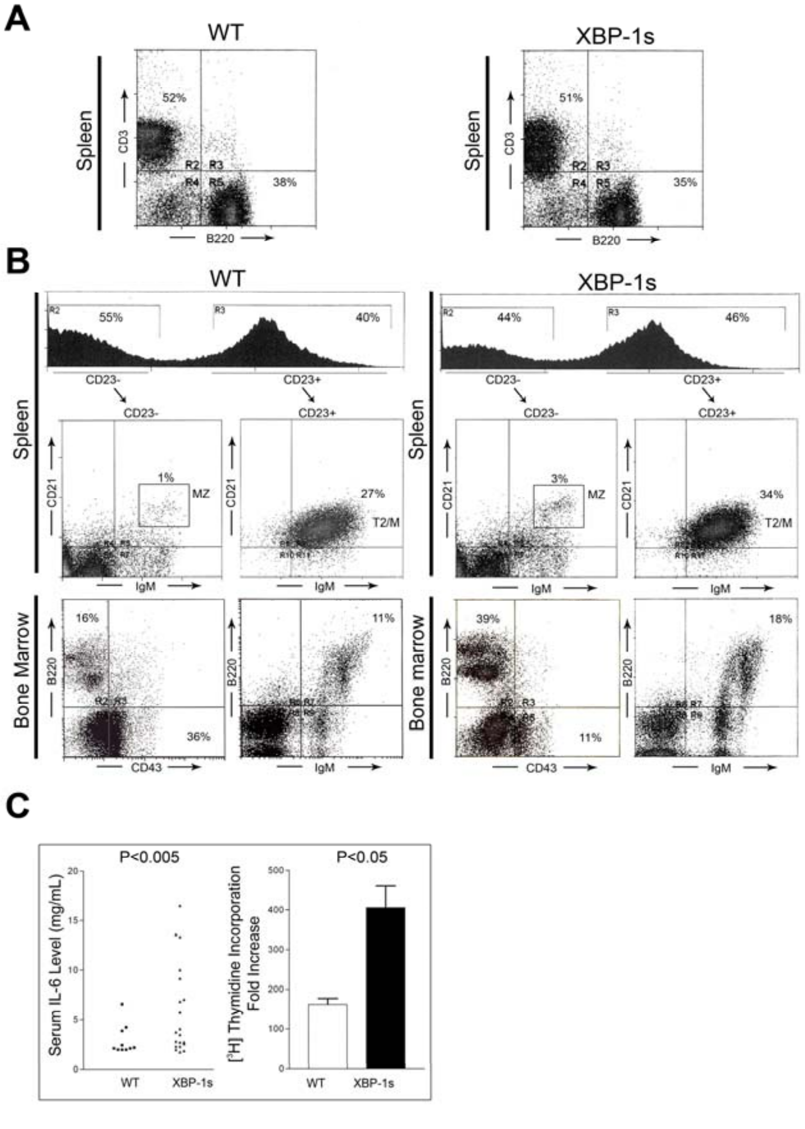

Figure S3. Altered Gene Expression of *Eμ-xbp-1s* B Cells and Tumor Plasma Cells

(A) Ingenuity analysis showing altered expression of CDBPA, CEBPB, CEBPD, and LITAF.

(B) Decreased MCL1 expression in *Eμ-xbp-1s* MM tumors based on the Affymetrix probe 1456381_x_at.

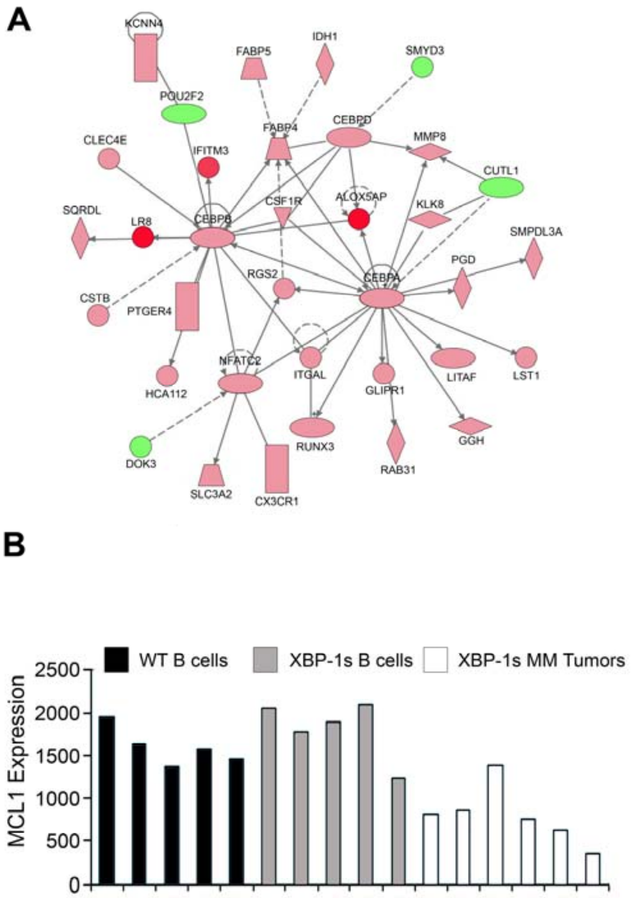

Table S1. Characterization of *Eu-xbp-1s* Transgenic Mice

No.	Age (Wks)	Strain	Age (Months)	Skin Alterations	Bone Lytic Lesions	M Spike	MGUS	MM	Isotype
1	30	S.7	8	+	-	-	-	-	
2	32	S.7	8	-	-	-	-	-	
3	32	S.7	8	+	ND	ND	-	-	
4	32	S.9	8	+	-	-	-	-	
5	32	S.9	8	-	-	-	-	-	
6	34	S.7	9	+	-	+	+	-	IgG,λ
7	34	S.7	9	+	ND	ND	-	-	
8	35	S.9	9	+	-	-	-	-	
9	37	S.7	9	+	-	-	-	-	
10	37	S.9	9	+	ND	ND	-	-	
11	38	S.7	10	-	-	-	-	-	
12	45	S.9	11	+	-	-	-	-	
13	45	S.9	11	+	ND	ND	+	-	polyclonal
14	45	S.7	11	+	ND	ND	-	-	
15	47	S.7	12	+	-	-	-	-	
16	47	S.9	12	+	-	+	+	-	polyclonal
17	48	S.9	12	+	-	-	-	-	
18	52	S.9	13	-	-	+	+	-	IgG,κ
19	56	S.7	14	+	-	-	-	-	IgM,λ
20	57	S.9	14	+	-	+	+	-	IgG,κ
21	58	S.9	15	-	-	-	-	-	
22	58	S.7	15	+	-	+	+	-	IgM,λ
23	59	S.7	15	-	+	+	-	+	IgM,κ
24	59	S.9	15	+	ND	ND	-	-	
25	60	S.7	15	-	-	-	-	-	
26	60	S.7	15	+	ND	ND	+	-	IgG,κ
27	60	S.7	15	+	+	+	-	+	IgM,κ
28	78	S.9	20	-	-	ND	+	-	IgM,κ
29	86	S.9	22	+	-	ND	+	-	IgG,κ
30	87	S.7	22	-	ND	ND	-	+	ND
31	90	S.7	23	-	+	ND	-	+	IgM,κ
32	91	S.9	23	-	ND	ND	-	+	IgG,κ
33	101	S.7	24	+	+	ND	-	+	IgG,κ
34	101	S.9	24	+	ND	ND	-	+	ND
35	102	S.7	24	-	+	ND	-	+	IgM,κ
Total				24/35	5/25	7/20	9/35	8/35	
Percentage				65%	20%	35%	26%	26%	

Clinical and histopathologic characterization of *Eμ-XBP-1s* transgenic mice. The disease manifestations presented in the table are of the propagated lines S7 and S9; however, similar disease was also documented in all transgenic founders.

Diagnostic criteria: MGUS: Presence of clonal M spike in the plasma, ≥10% clonal plasma cells in the bone marrow (BM), and absence of bone lytic lesions. MM: Presence of clonal M spike in the plasma, >10% of clonal plasma cells in the bone marrow, and presence of bone lytic lesions. Polyclonal: polyclonal serum hypergammaglobulinemia.
